# Sustained Reperfusion after Blockade of Glycoprotein-Receptor-Ib in Focal Cerebral Ischemia: An MRI Study at 17.6 Tesla

**DOI:** 10.1371/journal.pone.0018386

**Published:** 2011-04-01

**Authors:** Mirko Pham, Xavier Helluy, Christoph Kleinschnitz, Peter Kraft, Andreas J. Bartsch, Peter Jakob, Bernhard Nieswandt, Martin Bendszus, Guido Stoll

**Affiliations:** 1 Department of Neuroradiology, University of Heidelberg, Heidelberg, Germany; 2 Department of Experimental Physics, Section V, University of Würzburg, Würzburg, Germany; 3 Department of Neurology, University of Würzburg, Würzburg, Germany; 4 Rudolf-Virchow-Center, DFG Research Center for Experimental Biomedicine and Chair of Experimental Medicine, University of Würzburg, Würzburg, Germany; Charité Universitaetsmedizin Berlin, Germany

## Abstract

**Background:**

Inhibition of early platelet adhesion by blockade of glycoprotein-IB (GPIb) protects mice from ischemic stroke. To elucidate underlying mechanisms in-vivo, infarct development was followed by ultra-high field MRI at 17.6 Tesla.

**Methods:**

Cerebral infarction was induced by transient-middle-cerebral-artery-occlusion (**t**MCAO) for 1 hour in C57/BL6 control mice (N = 10) and mice treated with 100 µg Fab-fragments of the GPIb blocking antibody p0p/B 1 h after **t**MCAO (N = 10). To control for the effect of reperfusion, additional mice underwent permanent occlusion and received *anti-GPIb* treatment (N = 6; **p**MCAO) or remained without treatment (N = 3; **p**MCAO). MRI 2 h and 24 h after MCAO measured cerebral-blood-flow (CBF) by continuous arterial-spin labelling, the apparent-diffusion-coefficient (ADC), quantitative-T2 and T2-weighted imaging. All images were registered to a standard mouse brain MRI atlas and statistically analysed voxel-wise, and by cortico-subcortical ROI analysis.

**Results:**

*Anti-GPIb* treatment led to a relative increase of postischemic CBF vs. controls in the cortical territory of the MCA (2 h: 44.2±6.9 ml/100 g/min versus 24 h: 60.5±8.4; p = 0.0012, F_(1,18)_ = 14.63) after **t**MCAO. Subcortical CBF 2 h after **t**MCAO was higher in *anti-GPIb* treated animals (45.3±5.9 vs. controls: 33.6±4.3; p = 0.04). In both regions, CBF findings were clearly related to a lower probability of infarction (Cortex/Subcortex of treated group: 35%/65% vs. controls: 95%/100%) and improved quantitative-T2 and ADC. After **p**MCAO, *anti-GPIb* treated mice developed similar infarcts preceded by severe irreversible hypoperfusion as controls after **t**MCAO indicating dependency of stroke protection on reperfusion.

**Conclusion:**

Blockade of platelet adhesion by *anti-GPIb*-Fab-fragments results in substantially improved CBF early during reperfusion. This finding was in exact spatial correspondence with the prevention of cerebral infarction and indicates in-vivo an increased patency of the microcirculation. Thus, progression of infarction during early ischemia and reperfusion can be mitigated by anti-platelet treatment.

## Introduction

Ischemic stroke is a major cause of death and disability [1]. A significant proportion of strokes are caused by thromboembolic occlusion of major intracerebral vessels such as the middle cerebral artery (MCA). The complex cellular and molecular processes underlying the development of ischemic brain lesions are incompletely understood [2], [3]. This also applies to the situations in which extended and clinically severe strokes evolve despite “favorable” removal of the vessel occluding clot either spontaneously or by thrombolysis giving rise to reperfusion [4], [5]. Reperfusion is a prerequisite for replenishing brain areas at risk for infarction with oxygen and nutritional factors, but, on the other hand, elicits detrimental processes referred to as *reperfusion injury*. We could recently show that interference with critical steps of platelet tethering to the vessel wall can prevent ischemic stroke in the mouse model of transient MCA occlusion (**t**MCAO) [6], [7].The initial tethering of platelets at sites of vascular injury is mediated by GPIb-V-IX, a structurally unique receptor complex exclusively expressed in platelets and megakaryocytes [8]. GPIbα is indispensable for platelet adhesion under conditions of high shear such as in the arterial cerebrovascular system. Inhibition of the von Willebrand factor (vWF)-binding site of GPIbα with Fab fragments of the antibody p0p/B in wild-type mice abrogated platelet tethering and adhesion in a model of mechanically induced arterial thrombosis as well as in ischemic stroke, while unspecific Fab fragments had no effect [7]. Cerebral infarcts were significantly smaller when assessed histologically or by 1.5T MRI. These findings make GPIb an attractive target for clinical development of an antithrombotic drug in acute stroke.

There is recent evidence that GPIb, so far mainly regarded as instrumental in haemostasis and thrombus formation, can profoundly guide inflammation [9]. Thus, the effect of GPIb blockade in cerebral ischemia could be due to sustained patency of blood vessels during reperfusion or, alternatively, due to a primary anti-inflammatory effect [10]. To address this important issue, we employed multimodal MRI at ultra-high-field strength (UHF-MRI) to monitor lesion development in relation to cerebral blood flow in GPIb-Fab-treated mice and naive controls after **t**MCAO. As principal finding, we show that cerebral perfusion after **t**MCAO is sustained in GPIb-treated mice after removal of the vessel occluding thread while in normal mice perfusion further decreases leading to progressive stroke. Thus, *anti-GPIb* treatment substantially ameliorates infarct progression during early ischemia and reperfusion.

## Materials and Methods

### Experimental design and animal stroke model

All procedures and animal studies were approved by the Regierung von Unterfranken (Wuerzburg, Germany, approval number: 55.2-2531.01-23/04 and -55/09) and conducted in accordance with the recommendations for the performance of basic experimental stroke studies as previously published [11].

The main experimental group in this study were anti-GP1b treated mice (adult male C57/BL6 mice weighing 20–25 g (Charles River, Sulzfeld, Germany) undergoing one hour of **t**MCAO (N = 10). These mice received 100 µg p0p/B Fab fragments [12] intravenously 1 hour after **t**MCAO, that is, after 1 hour of occlusion at the time point at which the thread was removed. This regimen led to significantly smaller infarcts compared to control-treated animals in our previous study [7]. In the present study we used naive mice (adult male C57/BL6 mice weighing 20–25 g (Charles River, Sulzfeld, Germany) as controls for the efficacy to induce full-blown MCA infarcts by 1 hour of **t**MCAO(N = 10) because in our previous study there was no difference between naive mice and mice treated with an unspecific Fab fragment [7]. To investigate whether reperfusion is required for the therapeutic effect of GPIb blockade, additional anti-GPIb treated mice (N = 6) and control mice (N = 3) underwent permanent MCAO (**p**MCAO). Furthermore, another group of control mice underwent sham operation (N = 3).

The experimental procedures were performed as described in detail previously [7], [13], [14]. Briefly, a standardized suture coated with silicon rubber (6021PK10; Doccol Company, Redlands, CA, USA) was introduced into the right common carotid artery and advanced over the internal carotid artery to the origin of the MCA. The suture was fixed and left in situ and animals were allowed to recover. Operation time per animal did not exceed 15 minutes. After 60 min. animals were re-anesthetized and the suture was withdrawn to allow tissue reperfusion (**t**MCAO). For **p**MCAO, the thread was left within the vessel until the end of the experiments at day 1. Sham operation included preparation of the common carotid artery and ligation of its branches without thread insertion. The operations were performed under inhalation anesthesia (2.0% isoflurane in a 70%/30% N_2_O/O_2_ mixture) and the body temperature was maintained at 37°C using a servo-controlled heating pad. All subjects were subsequently followed in-vivo by serial multimodal UHF-MRI at 2 h and 24 h.

An additional group of *anti*-GP1b treated mice was investigated at an even earlier time point after tMCAO, i.e. 1 h after thread removal, to address the question whether the observed hypoperfusion at 2 h is preceded by hyperperfusion. In this scenario, a deleterious effect of reperfusion on tissue fate (reperfusion injury) might be functional rather than a beneficial effect of sustained reperfusion for the prevention of infarct progression under *anti*-GP1b treatment. In our local experimental setting of multimodal UHF-MRI, logistic circumstances restrict the earliest time point applicable for data acquisition to around 1 h after removal of the thread.

### Multimodal UHF-MRI of experimental cerebral ischemia in-vivo

A detailed description of the imaging protocol is given in previous work [15]. Cerebral perfusion was measured using a modified arterial spin labeling (CASL) method [16], [17], [18]. To benefit especially from increased longitudinal magnetization and the elevation of the T1 relaxation time for detailed anatomical mapping of CBF and group analysis, all measurements were performed at ultra-high magnetic field strength (Avance 17.6T, 750 MHz, Bruker BioSpin GmbH, Ettlingen, Germany). Image maps of cerebral perfusion were calculated on a pixel-by-pixel basis according to Detre et al. [18]. The degree of the inversion efficiency was assumed to be alpha = 0.7 [19], [20]. In close approximation to the value recently reported by Leithner et al. for the mouse brain [21] the brain-blood partition coefficient value for water was assumed to be lambda = 0.90 mL/g. Slice selective T1 mapping was measured with a single slice partial saturation inversion recovery RARE sequence (TI of 0.02 s, 0.5 s, 1.0 s, 1.5 s, 2.0 s, 3.0 s, 5.0 s, 10.0 s). The recovery time after acquisition of each image was 10 s (echo-train-length = 16, TE_eff_ = 30 ms). Inversion of magnetization was performed by a 6 mm slice selective adiabatic hypersecant pulse. T1 relaxation time constants were calculated voxel-wise applying first, a 3 parameter fit to estimate the efficiency of the inversion pulse and then, a 2 parameter fit with a fixed averaged value for the inversion pulse efficiency typically between 95% and 97%.

Diffusion weighted imaging (DWI) was performed with a pulsed-field gradient Setjskal-Tanner-like multislice spin echo sequence because echo-planar-imaging suffers from extreme susceptibility artifacts at ultra-high magnetic field strength. Diffusion sensitization was only performed along the slice direction to keep the overall acquisition time low [22]. Images with different b-values, 0 and 800 s/mm^2^, were acquired to allow for the calculation of apparent diffusion coefficient (ADC) maps of brain water. Whole brain coverage was achieved by thirteen coronal slices acquired with a matrix size of 64×64, FOV 1.8×1.8 cm, in plane resolution 281×281 µm, slice thickness = 0.5 mm, interslice distance = 1 mm, TE/TR = 22.3/2000 ms. Repeated measurement of the b = 800 s/mm^2^ DWI experiments (number of repetition NR = 3) led to an overall acquisition time for diffusion weighted experiments of 8 min. ADC maps were calculated by applying the common equation ADC = −0.00125×ln (SI_B800_/SI_B0_). The b value of 800 s/mm^2^ was chosen to maintain a high signal-to-noise ratio for each acquisition, in case motion artifacts of the spin echo DWI sequence would degrade other acquisitions.

T2 relaxometric mapping was performed for the in-vivo delineation of infarcted brain tissue at 24 h. Single slice T2-weighted (T2-w) imaging was performed using a Carr-Purcell-Meiboom-Gill (CPMG) multi-spin echo sequence collecting thirty two echoes at TR/TE = 4.2/2000 ms. T2 relaxation times constants were calculated voxel-wise by fitting the intensities of the 20 first echoes to a monoexponential model. CBF, T1 and T2 relaxometric maps were measured each with the exact same geometry for a 1.5 mm thick slab centered at the bregma as the operational definition of the central MCA territory.

For high-resolution structural imaging with whole-brain coverage, an additional strongly T2 weighted 2D turbo spin-echo sequence was acquired (RARE factor 16, TR = 8 s, effective TE = 56.44 ms, 2 averages, 13 coronal slices with an image matrix of 128×128 were acquired, FOV = 1.8 cm×1.8 cm, slice thickness = 0.5 mm, interslice distance = 1 mm, overall acquisition time of 2 min).

At the host console measurements and data processing were performed with the ParaVision software (version 3.02, Bruker BioSpin GmbH, Ettlingen, Germany). Further image calculation and fitting procedures were done using *MATLAB*® (The Mathworks Inc., Natick, MA, USA).

During UHF-MRI measurements mice were anesthesized by 2.0% Isoflurane in medical air (21%). The respiratory rate was monitored using an air-balloon positioned ventrally underneath the mouse body. The body temperature was constantly measured on the body surface and actively maintained at 37°C.

### Statistical and image analysis

The extraction of brain tissue from the scalp and skull was done by manual segmentation for each subject and time point. Packages from the FMRIB Software Library FSL (version 4.1) [23] were used for motion correction, registration (FLIRT) [24] and statistical image analysis. Intra-subject linear alignment and registration to a common standard template [25] was achieved by a step-wise affine procedure with six degrees of freedom. For voxel-wise statistical analyses, the global CBF maps were normalized by the overall average CBF value of the contralateral hemisphere.

CBF values early (at 2 h) and at 24 h after the experimental procedure were analysed for statistically significant voxelwise changes (24 h vs. 2 h) within the framework of the General Linear Model and corrected for multiple comparisons by nonparametric permutation testing using randomise, part of the FSL software library [23]. Randomise implements the method of permutation testing based on randomisation to correct for the multiple comparisons involved in testing across all image voxels to adequately protect against false-positive detections as described in detail by Nichols and Holmes [26]. For quantitative group comparisons, selected regions-of-interest (ROIs) were delineated in atlas space: 1) the cerebral cortex in the center of the MCA territory 2) the subcortex including the ipsilateral caudoputamen and pyramidal tract. Statistical analysis of ROIs was done by a 2×2 repeated measures ANOVA with factors of *GROUP* (**t**MCAO anti-GPIb; **t**MCAO controls) between-subjects, and *TIME* (2 h; 24 h) within-subjects. Additional groups to control for the experimental procedure (Sham controls, N = 3) and to control for recanalization and reperfusion (**p**MCAO anti-GPIb, N = 6) were analyzed separately. The risk of cerebral infarction was determined on within-group probability maps by averaging binary segmentations results of healthy vs. infarcted brain tissue within-subject. For each animal binary segmentation of cerebral infarction was performed in an automated fashion by applying a threshold of 34 ms T2 relaxation time on the T2 relaxometric maps at 24 h. Among different segmentation results for stepwise increasing T2 relaxation times, this cut off showed best agreement with visual delineation of infarction on T2-w imaging and with histological 2,3,5-triphenyltetrazolium chloride stain in selected subjects. Manual input was given only for the removal of intraventricular CSF. In addition, whole-brain volumetric analysis of infarcted tissue was retrieved by manual segmentation on T2-w RARE images.

## Results

### Cerebral perfusion in naive controls and *anti-GPIb* treated mice after transient and permanent MCAO

In naive control mice hypoperfusion extended over cortical and subcortical ROI's in the center of the MCA territory at 2 h and was followed by a significant further decrease in CBF at 24 h after **t**MCAO (cortical CBF (ml/100 mg/min): 40.9±4.4 (2 h) vs. 26.0±3.2 (24 h), p = 0.022; subcortical CBF: 33.6±4.3 (2 h) vs. 24.8±3.2 (24 h), p = 0.009). In contrast, *anti-GPIb* treated mice showed significant reperfusion of the cortex (44.2±6.9 (2 h) vs. 60.5±8.4 (24 h), p = 0.037). In the subcortex, initial CBF of the *anti-GPIb* group was higher than in controls (33.6±4.3 (controls at 2 h) vs. 45.3±5.9 (*anti-GPIb* at 2 h), p = 0.047). Subcortical CBF remained stable at 24 h in *anti-GPIb* treated mice (46.9±7.5) but further declined in controls (24.8±3.2). Table 1 gives an overview of mean CBF values within cortical and subcortical ROI's in the center of the MCA territory.

**Table 1:** All outcome measures per group, time point and location.

		Controls	Anti-GPIb
**CBF (ml/100 mg/min) 2 h vs. 24 h**	**Cortex**	40.9±4.4 vs. 26.0±3.2	44.2±6.9 vs. 60.5±8.4
	**Subcortex**	33.6±4.3 vs. 24.8±3.2	45.3±5.9 vs. 46.9±7.5
ADC (mm^2^/s*10^−4^) 2 h vs. 24 h	**Cortex**	6.48±0.27 vs. 5.75±0.23	7.88±0.28 vs. 7.53±0.26
	**Subcortex**	6.08±0.60 vs. 5.29±0.33	7.86±0.33 vs. 7.12±0.26
**qT2 (ms) 2 h vs. 24 h**	**Cortex**	37.24±1.96 vs. 60.05±3.15	28.6±0.4 vs.29.0 ±0.97
	**Subcortex**	33.41±1.05 vs. 49.89±3.15	30.6±0.3 vs. 37.4±2.2
**Probability of Infarction (%) 2 h vs. 24 h**	**Cortex**	60.9±9.3 vs. 95.1±2.8	17.4±2.1 vs. 34.5±8.1
	**Subcortex**	79.1±9.9 vs. 100±0	21.5±7.9 vs. 64.8±14.5

Values are expressed as group means and corresponding standard errors. As the main finding sustained reperfusion was observed in *anti-GPIb* treated mice, whereas controls exhibited significant progression of hypoperfusion from 2 h to 24 h. In the cortex of the MCA territory, reperfusion significantly increased from 2 h to 24 h in *anti-GPIb* treated mice presumably related to a larger capacity of collateral blood flow as compared to the subcortex. In the subcortex of *anti-GPIb* treated mice, improved reperfusion as compared to controls was reflected by a significantly higher baseline CBF at 2 h. Sustained reperfusion both in the cortical and subcortical territory of the MCA was associated with a protection from cerebral infarction as evident by a low probability of infarction.

**Figure 1:**
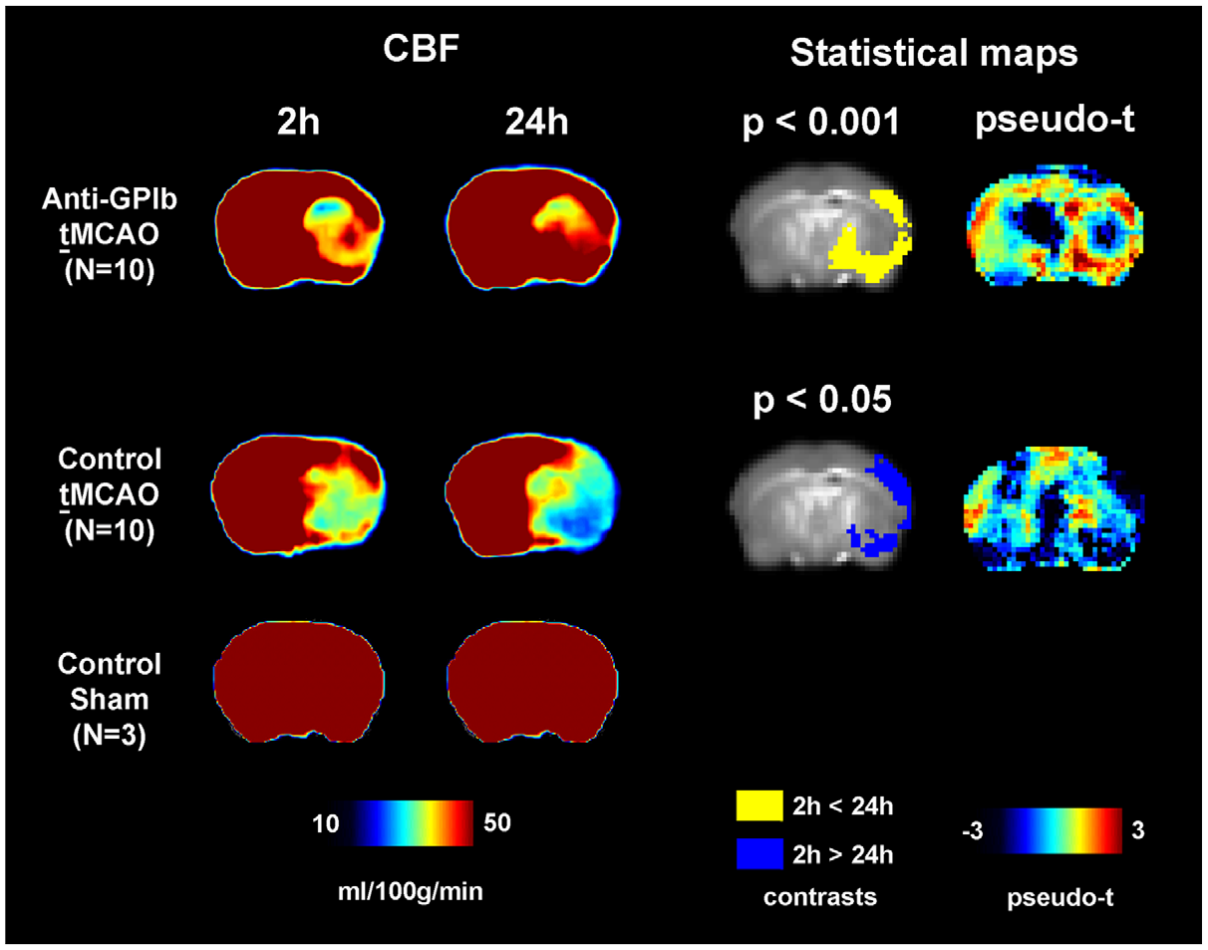
CBF and statistical maps of voxel-wise group comparisons. Sustained reperfusion is demonstrated by significantly elevated cortical perfusion in *anti-GPIb* treated mice as compared to persisting severe hypoperfusion in control mice. Color maps of mean CBF are given for each group and time point (left, CBF). The results of voxel-wise statistical analyses of change in CBF over time are shown on the statistical parameter maps (right, Statistical maps). The spatial distribution of significant reperfusion in the *anti-GPIb* treated group is indicated by the yellow overlay (yellow contrast of 24 h>2 h). The spatial distribution of significant deterioration of hypoperfusion in control mice after **t**MCAO is indicated by blue overlay (blue contrast of 2 h>24 h).

Correspondingly, on voxel-wise analysis, clusters of significant perfusion activation (reperfusion) and deactivation (deterioration of hypoperfusion) were found. In *anti-GPIb* treated mice reperfusion was located in the cortex, mainly in the distribution of the middle and posterior cerebral artery. In control mice, however, hypoperfusion deteriorated in the center of the cortical territory of the middle cerebral artery and in a smaller temporobasal cluster in the distribution of the posterior cerebral artery. Figure 1 shows the location of significant clusters of perfusion activation/deactivation in standard space (blue overlay for the contrast 2 h>24 h; yellow overlay for the contrast 24 h>2 h).

In contrast, *anti-GPIb* treated mice with permanent vessel occlusion (**p**MCAO) experienced progression of severe hypoperfusion (2 h: 42.9±11.5 vs. 24 h: 35.4±5.2) and developed extended complete MCA infarctions (not shown) similar to naive controls. This indicates that *anti-GPIb* treatment is ineffective after permanent vessel occlusion. Sham operated control mice (N = 3) did not exhibit any perfusion abnormalities at 2 h or 24 h after the experimental procedure and did not develop cerebral infarctions (Figure 1).

In line with the results at 2 h and 24 h, increased cortical CBF was also observed 1 h after tCMAO (1 h: 28.2±3.5 ml/100 g/min vs. 24 h: 110.09±10.0; n = 4/group; p = 0.002). This effect was still robust when evaluating CBF ratios between ipsilateral and contralateral mirror ROIs: (1 h: 0.19±0.01 vs. 24 h: 0.56±0.06; p = 0.005). In addition, quantitative cortical and subcortical T2 values (ms) representing infarct probability in these areas were similar in comparison with the original group of *anti*-GP1b treated mice measured at 2 h and 24 h (cortical ROI 1 h: 30.6±0.7 vs. cortical ROI 24 h: 32.2±2.1; subcortical ROI 1 h: 30.8±0.3 vs. subcortical ROI 24 h: 45.7±2.4).

### Probability of cerebral infarction and quantitative ADC values in naive and *anti-GPIb* treated mice

**Figure 2:**
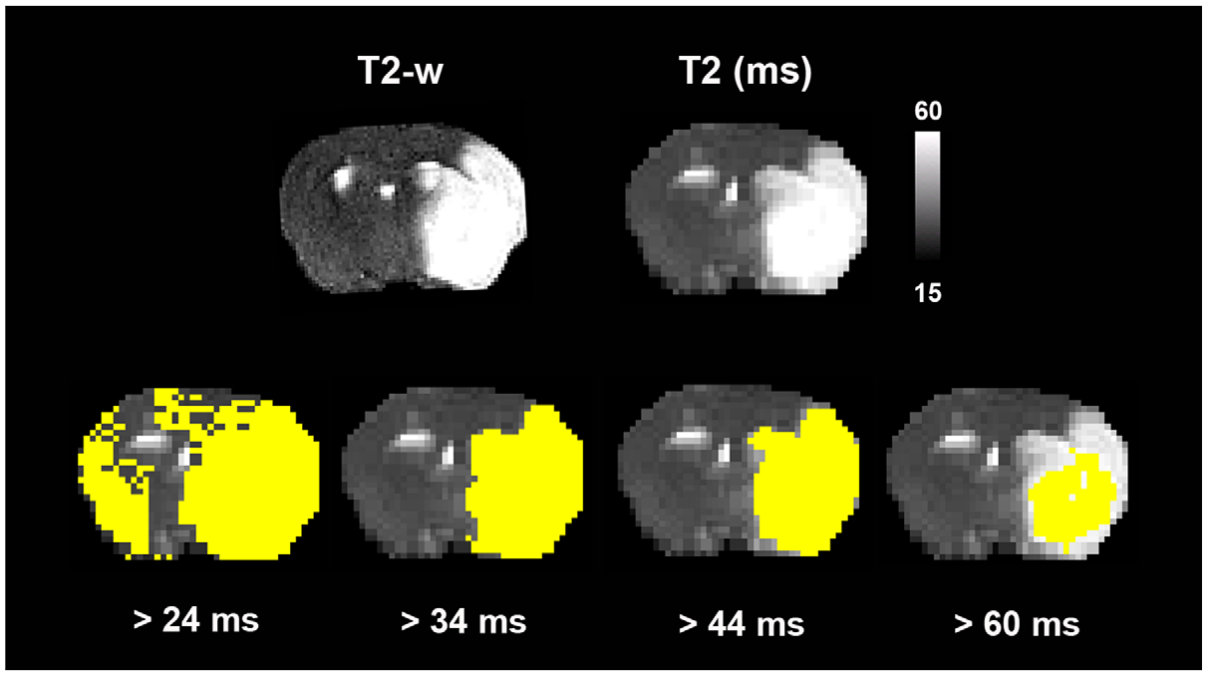
Results of automated stepwise binary segmentation of cerebral infarction. Different segmentation results are displayed for increasing quantitative T2 values. Accurate estimates of the extent of cerebral infarction as compared to high-resolution T2-w imaging (upper left) and histological stains with 2,3,5-triphenyltetrazolium chloride were achieved for a segmentation threshold of T2 = 34 ms. Delineation of infarction was performed on T2 relaxometric images at 24 h after the experimental procedure.

Cerebral infarction was determined on T2 relaxometric images by binary segmentation at a threshold of 34 ms. This cut-off was previously demonstrated to give accurate estimates of infarct extension at 17.6 Tesla field strength [15]. Figure 2 shows that the cut-off value of T2 = 34 ms yields best results of infarct extension when comparing the results of a stepwise segmentation procedure with increasing quantitative T2 thresholds as compared to high resolution T2-w RARE imaging.

**Figure 3:**
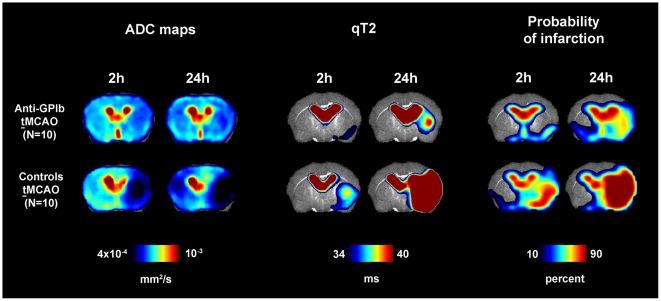
Maps of ADC (left), quantitative T2 (qT2, middle) and probability of infarction (right). Measures are given as group means per time point. The lower threshold of qT2 was chosen to be the segmentation threshold for infarction (34 ms). Strong protection from cerebral infarction was observed in *anti-GPIb* treated mice reflected by a substantially lower probability of completed infarction as evident on ADC, qT2 and probability maps (upper row, *anti-GPIb*). This finding was most marked in the cortex of the MCA territory. In control mice after **t**MCAO, cerebral infarction involved the cortex in the center of the MCA territory and the complete deep MCA territory with high probability (lower row, controls **t**MCAO).

Probability maps of cerebral infarction were rendered group-wise using the individual segmentation results of each animal. They are given along with maps of the mean ADC for each group and time point in Figure 3. Of note, for the given segmentation threshold, in control mice after **t**MCAO cerebral infarction was already manifest at 2 h in the basal ganglia and covered the complete cortical and deep MCA territory at 24 h. In *anti-GPIb* treated mice infarction did not occur with relevant probability at 2 h after **t**MCAO (17.4±2.1%) and at 24 h occurred with a significantly lower probability in the cortex and basal ganglia than in corresponding ROI's in controls (cortex: 34.5±8.1% vs. 95.1±2.8%, p = 0.0001; subcortex: 64.8±14.5% vs. 100±0%, p = 0.01). Quantitative T2 values showed similar group differences. Cortical and subcortical quantitative ADC values exhibited a significantly stronger decrease in controls than in *anti-GPIb* treated mice (cortex: p = 0.001; subcortex: p = 0.003). Table 1 displays all values of ADC, quantitative T2 and probabilities of infarction for each group, ROI and time point.

**Figure 4:**
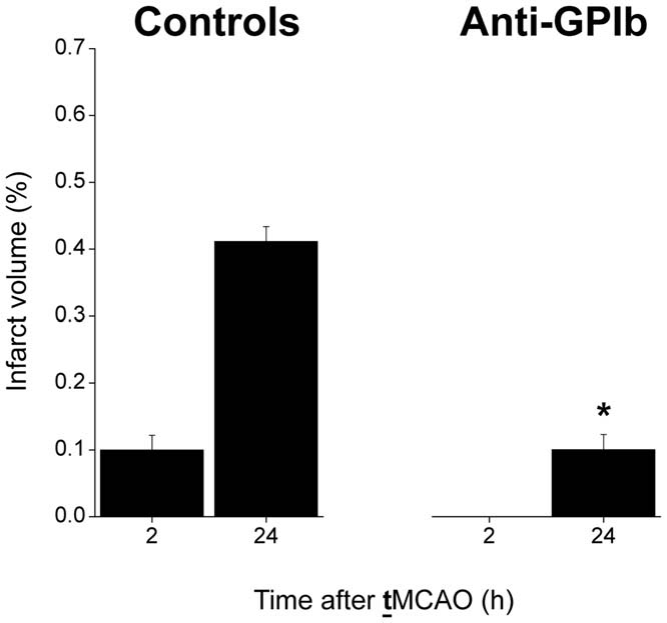
Whole-brain volumetric analysis of cerebral infarction. The whole-brain segmentation of cerebral infarction was accomplished by visual delination on T2-w RARE images. The infarct volume is given as percentage of the whole-brain volume. Control mice after **t**MCAO (left) showed extended cerebral infarctions in the MCA territory at 24 h, preceded by smaller subcortical infarctions at 2 h. In contrast, *anti-GPIb* treated mice exhibited significantly smaller volumes of completed infarctions (*anti-GPIb* vs. controls at 24 h, *p = 0.0002). In addition, early after **t**MCAO at 2 h, cerebral infarction was not detected on visual analysis corresponding to a neglibly small probability of infarction as detected by automated segmentation (see Figs. 3 and 5).

Whole brain volumetric measurement of cerebral infarction by manual delineation was performed additionally and showed similar group differences between control mice and *anti-GPIb* mice after **t**MCAO as observed by automated segmentation in the center of the MCA territory (Figure 4).

Intracerebral hemorrhage was not observed in any of the anti-GPIb treated mice which is in accordance with our previous study [7].

### Comprehensive group analyses of CBF response from cortical and subcortical regions-of-interest

**Figure 5:**
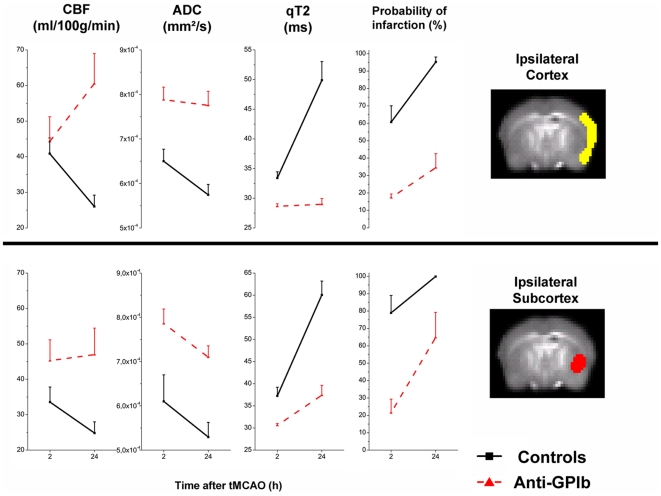
Plots of all outcome measures within cortical (upper half) and subcortical (lower half) ROI's per group and time point. Control mice after **t**MCAO (solid black lines) and *anti-GPIb* treated mice (dashed red lines) showed significant group differences in the postischemic course of CBF (first column): sustained reperfusion in *anti-GPIb* treated mice vs. progression of hypoperfusion in controls, which was most marked in the cortex (Interaction GROUPxTIME; p = 0.00001). This effect of CBF was paralleled by a decrease in cortical and subcortical ADC in controls, but stable cortical ADC in *anti-GPIb* treated mice and less severe subcortical ADC decrease in *anti-GPIb* treated mice as compared to controls (second column). The third column (qT2) indicated severe increase in the cortical and subcortical T2 relaxation constant over time reflecting the demarcation of irreversible tissue damage for controls. In contrast, *anti-GPIb* treated mice exhibit stable T2 values in the cortex protected from infarction and in the subcortex exhibit only moderate increase in mean T2 as compared to control mice consistent with a lower group probability of infarction in this region. Probability of infarction (fourth column) by automated segmentation of individual animals is consistently lower for *anti-GPIb* treated mice, a finding most marked in the cortex.

Outcome measures of cerebral perfusion (CBF) and completed cerebral infarction (qT2) were calculated from cortical and subcortical ROIs as indicated in atlas space and are plotted in Figure 5. The cortical ROI was associated with the ipsilateral cortical MCA territory (yellow overlay in ipsilateral cortex), the subcortical location comprised the ipsilateral caudoputamen and deep pyramidal tract (red overlay in ipsilateral subcortex).

Group comparison of CBF from the cortical ROI, similar to voxel-wise image analysis, demonstrated that the time course of CBF between both groups went in opposite directions showing deterioration in controls (2 h: 40.9±4.4; 24 h: 26.0±3.2) and strong recovery of CBF indicating sustained reperfusion in *anti-GPIb* treated mice (2 h: 44.2±6.9; 24 h: 60.5±8.5). This is reflected by the significant interaction between the factors *GROUP* and *TIME* in the repeated measures ANOVA (p = 0.0012, F_(1,18)_ = 14.63).

The significant main effect of improved cortical reperfusion in *anti*-GP1b treated mice in ipsilateral ROIs was still robust when CBF ratios between ipsilateral and contralateral mirror ROIs were evaluated (2 h: 0.31±0.070 vs. 24 h: 41±0.07; p = 0.01).

Group comparison of CBF from the subcortical ROI showed deterioration of severe hypoperfusion in naive controls (2 h: 33.6±4.4 and 24 h: 24.8±3.2). In contrast, sustained reperfusion was observed in *anti-GPIb* treated mice (2 h: 45.3±5.9 and 24 h: 46.9±7.5). The postischemic baseline value of subcortical CBF at 2 h was significantly lower in controls than in the *anti-GPIb* treated group (2 h: 33.6±4.4 vs. 45.3±5.9; p = 0.04).

Quantitative cortical T2 values were significantly different between groups (GROUP: F_(1,18)_ = 14.63, p<0.00001), both time points (TIME: F_(1,,18)_ = 35.83, p<0.00001) and also with a strong interaction between GROUP and TIME (GROUPxTIME: F_(1,,18)_ = 35.83, p<0.00001). All T2 measures are given in Table 1.

The additional group of N = 4 *anti*-GP1b treated mice investigated very early after tMCAO 1 h after thread removal also exhibited strong reperfusion, which was most marked in the cerebral cortex: 28.2±3.5 ml/100 g/min (cortex at 1 h) vs. 110.1±10.0 (cortex at 24 h); p = 0.002. This effect was robust against evaluating CBF ratios between ipsilateral and contralateral mirror ROIs: 0.19±0.01 (cortex at 1 h) vs. 0.56±0.06 (cortex at 24 h); p = 0.005. In this additional series, the observed quantitative T2 values (ms), and hence the probability of infarction, within the cortical (1 h: 30.6±0.7 vs. 24 h: 32.2±2.1) and subcortical ROI (1 h: 30.8±0.3 vs. 24 h: 45.7±2.4) were similar in comparison with the original group of *anti*-GP1b treated mice undergoing measurements at 2 h and 24 h.

## Discussion

This UHF-MRI study confirms and extends our previous observation that blockade of platelet tethering in experimental cerebral ischemia prevents infarct growth. Importantly, for the first time we show in-vivo that interfering with platelet function can prevent naturally occurring blood flow reductions in the brain during reperfusion. Thus, platelets are important mediators of infarct progression during ischemia and reperfusion.

GPIb-V-IX is a structurally unique receptor complex exclusively expressed in platelets and megakaryocytes which mediates the initial tethering of platelets at sites of vascular injury [8]. Blockade of GPIb ameliorates infarct growth in cerebral ischemia as shown previously [7], and confirmed here, but the underlying mechanisms are largely unknown. Tethering of platelets to the endothelial layer of the vessel wall via GPIb/vWF binding is an important initiator of thrombus formation under high shear conditions such as in the arterial system [3], [6], [8], [27]. On the other hand, blockade of GPIb can prevent immune cell recruitment in the context of inflammation as shown recently in a model of experimental peritonitis [9]. Since inflammation is more and more recognized as a critical component in the pathophysiology of stroke [28], [29], [30], it was conceivable that the beneficial effects of GPIb blockade on infarction are not related to improving blood flow, but rather to ameliorating secondary inflammation.

To address this important issue we employed multimodal UHF-MRI at 17.6T in mice with stroke allowing the measurement of cerebral blood flow in-vivo over time, through the intact skull and with extended anatomical sampling both of deeply located brain regions and the cortex. In addition, impending infarction was assessed by the measurement of hypoxic diffusion restriction of free water (ADC) and quantitative T2 was evaluated as a marker for vasogenic edema paralleling completed tissue infarction. All measures were acquired with high spatial resolution and extended coverage to achieve segmentation of the mouse cortex from subcortical structures. These brain regions show differences in their susceptibility to ischemia since the neocortex has a larger capacity for collateral blood supply. Data postprocessing and analysis included registration of all outcome measures into one anatomical standard space to enable voxel-wise statistical comparisons on group level and the calculation of probability maps of infarction for each group.

By means of calculating probability maps of infarction for each group, we could show that *anti-GPIb* treatment protects the cerebral cortex downstream of the recanalized MCA from infarction. There was a significantly lower probability of completed infarction in this region for *anti-GPIb* treated mice as compared to controls at 24 h after **t**MCAO (35% vs. 95% in controls, p = 0.0001). CBF measurements revealed that this was due to sustained reperfusion after *anti-GPIb* treatment. Treated mice showed a higher CBF already very early during reperfusion (at 1 h and 2 h after **t**MCAO) as compared to controls (subcortex: 45.3±5.9 vs. 33.6±4.3; cortex: 44.2±6.9 vs. 40.9±4.4) and this difference further increased with time (subcortex at 24 h: 46.9±7.5 vs. 24.8±3.2; cortex at 24 h: 60.5±8.4 vs 26.0±3.2). Sustained reperfusion was also observed in the basal ganglia, which, although to a lesser degree than the cortex, were also protected from infarction in comparison with controls. The fact that infarct protection was lost after permanent MCAO clearly indicates that recanalization of large proximal vessels is compulsory for the efficacy of anti-GPIb Fab fragments. The exact mode of anti-GPIb Fab action in the context of brain ischemia/reperfusion injury still has to be established but reduced thrombus formation or destabilisation of previously formed thrombi leading to improved clot “washout” from the cerebral microvasculature might be involved [15]. Since GPIb-V-IX is a receptor complex exclusively expressed in platelets and megakaryocytes, the stroke-mitigating effect by anti-GPIb Fab can only be explained by an anti-platelet effect, and not by direct neuroprotection that could secondarily improve blood flow.

As an additional exploratory finding, we observed a T2 increase already 2 h after tMCAO in control mice. This probably indicates very early vasogenic edema detectable only at ultra-high magnetic field strengths and is in accordance with measurements at 9.4 Tesla [31], [32].

Taken together, our study shows that preventing platelet activation can prevent deterioration of blood flow during the reperfusion phase after transient cerebral ischemia. Using multimodal in-vivo MRI at ultra-high magnetic field strength, wefor the first time could verify increased microvascular patency early during reperfusion in the wake of anti-GP1b treatment which is supported by previous histological data [33]. These results further support the concept that platelets play an important role in mediating infarct progression during early ischemia and successive reperfusion. Thus, GPIb, and its downstream signalling pathways via phospholipase D1 [34] may be promising new targets to combat acute ischemic stroke in the future.
